# Brief research report: Contrast-enhanced radiography for gonadal visualization in Nile tilapia

**DOI:** 10.3389/fvets.2026.1855809

**Published:** 2026-06-22

**Authors:** Mokhamad Fakhrul Ulum, Ali Omar Alahmad, Adelaide Jose Pereira Cristovao, Christian C. Santos, Fajar Maulana, Danang Dwi Cahyadi

**Affiliations:** 1 Division of Reproduction and Obstetrics, School of Veterinary Medicine and Biomedical Sciences, IPB University, Bogor, West Java, Indonesia; 2 Faculty of Veterinary Medicine, University of Hama, Hama, Syria; 3 Animal Health Department, Faculty of Agriculture, Universidade Nacional Timor Lorosa'e (UNTL), Dili, Timor-Leste; 4 College of Veterinary Science and Medicine, Central Luzon State University, Science City of Muñoz, Nueva Ecija, Philippines; 5 Division of Aquatic Organism Reproduction and Genetics, Department of Aquaculture, Faculty of Fisheries and Marine Sciences, IPB University, Bogor, West Java, Indonesia; 6 Division of Anatomy Histology and Embryology, School of Veterinary Medicine and Biomedical Sciences, IPB University, Bogor, West Java, Indonesia

**Keywords:** aquaculture, digital radiography, non-destructive imaging, *Oreochromis niloticus*, positive contrast, reproductive assessment, reproductive imaging, veterinary imaging

## Abstract

Evaluation of gonadal visualization is important for reproductive management in aquaculture. This study introduces a novel method using a positive contrast medium in digital radiography to enhance gonadal visualization in Nile tilapia (*Oreochromis niloticus*). The contrast medium was administered retrogradely via an intravenous catheter sheath before laterolateral and dorsoventral radiographic imaging. In well-developed males, the agent outlined the spermatic ducts and branching channels. In well-developed females, it highlighted the spaces between oocytes, appearing as rounded radiolucent granular structures within a radiopaque background. This method allows for the preliminary assessment of reproductive status, including gonadal development and potential fecundity. Positive contrast radiography enhances animal welfare and may support breeding efficiency, supporting its integration into aquaculture for sustainable reproductive management.

## 1 Introduction

Contrast agents enhance diagnostic imaging by improving organ delineation, aiding accurate diagnoses, and monitoring. In fish, the proximity of abdominal organs often causes superimposition on radiographs, hindering structure identification. Radiography has been used in fish for species identification (1), detecting foreign bodies and skeletal abnormalities (2), and internal organ visualization (3). Computed tomography also assists with skeletal assessments (2). Recently, aquatic veterinary imaging has expanded to multimodal approaches, including ultrasonography, CT, MRI, and virtopsy-based protocols, for evaluating coelomic and postmortem structures in non-mammalian species (4–6). Despite these advances, contrast-enhanced radiography for fish gonadal identification remains unexplored, and positive contrast agents are mainly used in gastrointestinal studies, especially oral barium sulfate for gastric transit and digestive evacuation (7, 8).

Accurate reproductive assessment in fish commonly relies on methods such as chemical staining of the genital papilla (9, 10), histology, laparoscopy (11–13), and postmortem fecundity estimation based on ovarian weight and oocyte density (14). These approaches, although informative, are invasive and often terminal, posing ethical and practical concerns for valuable broodstock and conservation-priority species. Non-destructive alternatives, such as ultrasonography, have been used to monitor gonadal development in silver eels (*Anguilla anguilla*) (15), lumpfish (*Cyclopterus lumpus*) (16), Nile tilapia (*Oreochromis niloticus*) (17), and common carp (*Cyprinus carpio*) (3); however, interpretation requires operator expertise and may be limited in smaller fish. Sex determination in Nile tilapia becomes difficult below 400 g because of the 1–2 mm thickness of the testes (17), with even larger body sizes required in rainbow trout (18). Building on previous observations that plain digital radiography can localize internal organs but fails to clearly define organ margins in common carp (3), this study evaluated the feasibility of retrograde positive contrast-enhanced radiography for visualizing gonadal boundaries and internal gonadal visualization in Nile tilapia and investigated whether it improves the differentiation of testes and ovaries compared with plain radiography as a preliminary non-lethal approach for reproductive assessment in fish.

## 2 Materials and methods

### 2.1 Animals

A total of 32 black and red Nile tilapia (*Oreochromis niloticus*) were studied, comprising 16 males (body weight 325.20 ± 94.58 g, body length 257.50 ± 37.38 mm) and 16 females (body weight 295.67 ± 85.23 g, body length 245.83 ± 18.55 mm). Healthy fish were purchased from a live fish vendor at a local market, and their gonadal maturity status was unknown. Physical examination revealed no external lesions or abnormalities, confirming that all fish were suitable for the study. All procedures complied with ethical standards for animal handling and welfare, ensuring appropriate use for research and were approved by the Animal Ethics Committee, IPB University (approval no. 321/KEH/SKE/IV/2025).

### 2.2 Animal handling and restraint

Before radiographic imaging, the fish were maintained in aerated laboratory freshwater and monitored for behavioral stability. The fish were immobilized using cold-water sedation with chilled water and ice cubes for approximately 1 h, until spontaneous movement decreased but opercular activity continued (3, 19). The body surface was gently dried with a chamois cloth to minimize artifacts, and radiographs were obtained in each projection before and after contrast administration for evaluation. Radiographic imaging was conducted in live fish after sedation (fresh) to prevent autolysis or gas artifacts from affecting the contrast medium or gonadal architecture clarity.

### 2.3 Digital radiography imaging

#### 2.3.1 Plain radiography

Digital radiography was performed using a POX-100BT mobile X-ray unit with a 100 cm focus-to-film distance, operated at 48 kV and 2.5 mAs. Nile tilapia were imaged dorsoventrally and laterally to visualize skeletal structures and internal organs in detail, with an emphasis on the gonads. Careful positioning ensured stability and optimal image quality, with styrofoam supports maintaining proper body alignment during dorsoventral imaging.

#### 2.3.2 Contrast radiography procedure

**Figure 1:**
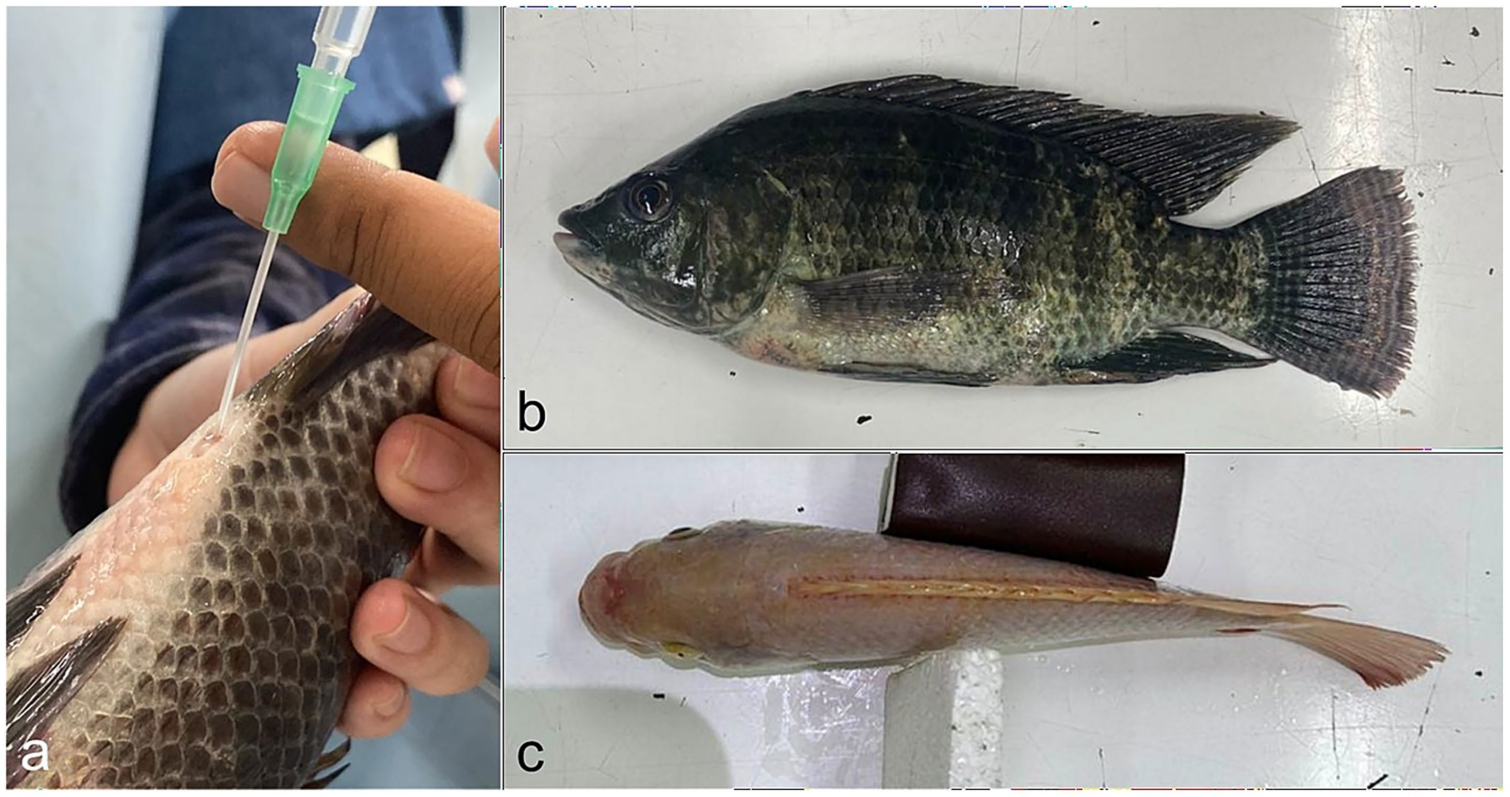
Retrograde administration of a radiographic contrast agent (Iopamidol, Iopamiro 300, Dipa Healthcare, Indonesia) in Nile tilapia (*Oreochromis niloticus*) through the genital opening using a plastic intravenous catheter sheath, with the fish positioned head-down for contrast flow into the reproductive tract. **(a)** Contrast agent administration using 22G IV catheter sheath, **(b)** lateral and **(c)** dorsoventral projections during digital radiography.

Iopamidol (Iopamiro 300, Dipa Healthcare, Indonesia) was administered retrogradely into the reproductive tract using an 18G or 22G IV catheter sheath inserted 5 mm into the genital opening at 1–2 mL. The volume was adjusted based on fish size and injection resistance. Smaller fish received less, while larger fish tolerated more. An 18G or 22G IV catheter sheath was selected according to fish size. The contrast medium was administered slowly to minimize pressure and reduce tissue damage or leakage. Administration stopped if resistance was encountered. No hemorrhage, gonadal rupture, or mortality was observed. Tilapia were positioned with heads slightly lowered to facilitate contrast medium flow, filling intercellular cavities in females or spermatic ducts in males (Figure 1). Excess contrast medium was removed with tissue paper to avoid contamination. Digital radiographs were obtained in dorsoventral and lateral projections to visualize the distribution of the contrast agent within the reproductive system.

### 2.4 Necropsy for validation of digital radiographic imaging

**Figure 2:**
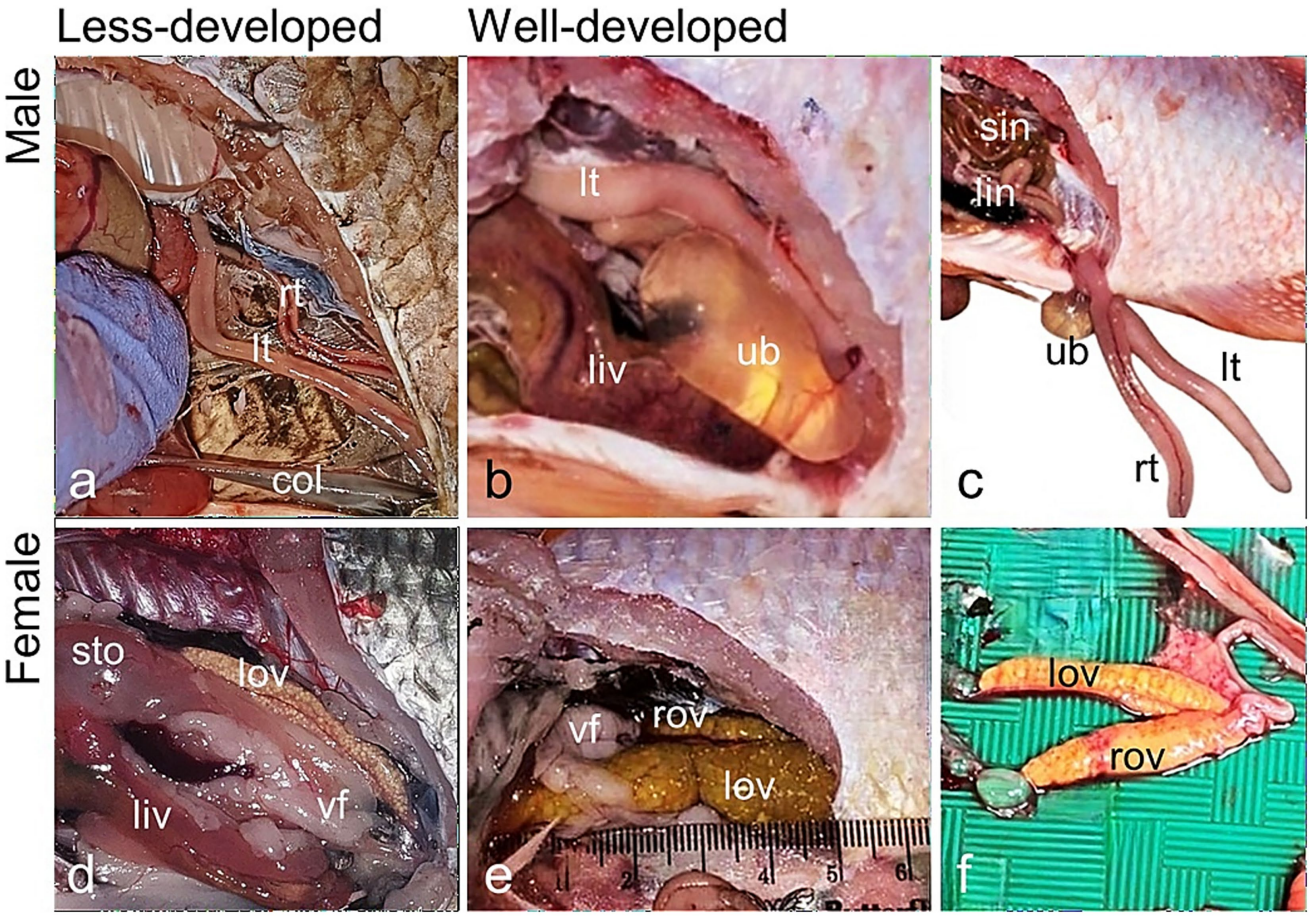
Necropsy findings in male and female Nile tilapia (*Oreochromis niloticus*). **(a)** Male tilapia with less-developed and **(b)** well-developed testes in the abdominal cavity; **(c)** exteriorized testes after incision; **(d)** female tilapia with less-developed and **(e)** well-developed ovaries in the abdominal cavity; **(f)** paired ovaries removed, containing yellow–orange oocytes in sacs. sto, stomach; liv, liver; vf, visceral fat; lin, large intestine; sin, small intestine; col., colon; lt, left testicle; rt., right testicle; ub, urinary bladder; lov, left ovary; and rov, right ovary.

Following gradual cooling according to established protocols (19), the fish were euthanized via cervical dislocation. During necropsy, the abdominal cavity was opened using scissors, and an incision was made from the anal region to the operculum (Figure 2). Muscle tissue was lifted to expose internal organs, allowing direct examination of anatomical structures to validate findings from digital radiographic imaging (20).

### 2.5 Data analysis

Digital radiographic images were analyzed to identify gonadal anatomical features of the fish. The necropsied organs were examined and correlated with radiographic images to validate the visualization accuracy.

## 3 Results

Based on radiographic appearance and necropsy confirmation, fish were categorized as having less-developed or well-developed gonads. However, this study did not establish a complete histological staging system. Therefore, “premature” and “mature” indicate differences in gross gonadal development and contrast distribution, not definitive histological maturity stages.

**Figure 3:**
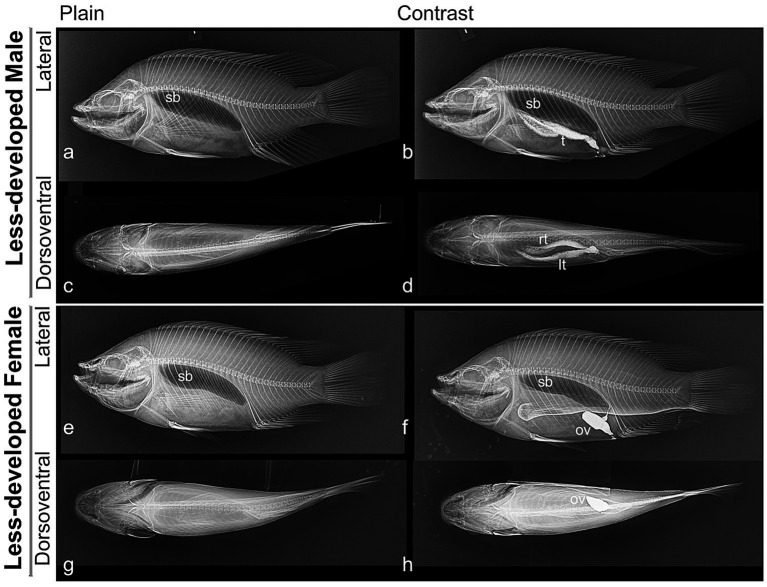
Plain and contrast-enhanced radiographs of less-developed Nile tilapia (*Oreochromis niloticus*) in laterolateral (LL) and dorsoventral (DV) projections. **(a,c,e,g)** Plain radiographs show the gonads as faint, slightly radiopaque silhouettes with poorly defined margins. **(b,d,f,h)** Contrast-enhanced radiographs show more radiopaque male and female gonads with clearly defined margins than plain radiographs. sb, swim bladder; t, testicle; rt., right testicle; lt, left testicle; ov, ovary.

Figure 3 illustrates plain and contrast-enhanced radiographic images of Nile tilapia (*Oreochromis niloticus*) obtained in lateral and dorsoventral projections. In plain radiographs, the gonads appeared faint and were only weakly discernible beneath the swim bladder. In contrast-enhanced imaging, clear delineation of both left and right reproductive organs—either testes or ovaries—was achieved, allowing accurate assessment of gonadal margins and size. In the dorsolateral view, the paired gonads were symmetrically visualized along the body axis within the abdominal cavity, whereas the laterolateral projection demonstrated their anatomical position beneath the kidneys and swim bladder and directly above the gastrointestinal tract.

In males, the contrast agent outlined the spermatic ducts within each testis as fine, branching radiopaque channels converging toward the spermatic tract and ultimately opening at the urogenital pore. Following positive contrast administration, the gonads of less-developed male tilapia located in the medial abdominal cavity appeared more radiopaque, forming a characteristic Y-shaped pattern extending from the caudal to the cranial region of the abdomen. In contrast, less-developed female gonads appeared as highly radiopaque tissue concentrated in the ventro-caudal portion of the abdominal cavity.

**Figure 4:**
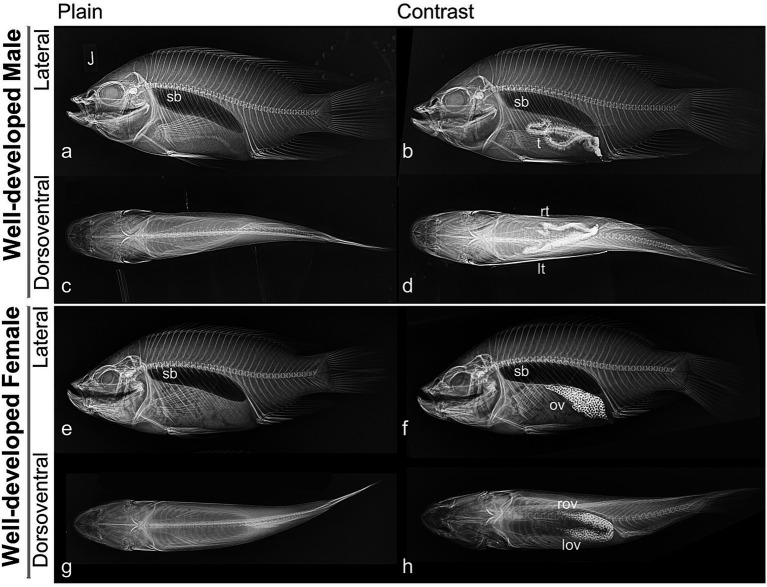
Plain and contrast-enhanced radiographs of Nile tilapia (*Oreochromis niloticus*) in laterolateral (LL) and dorsoventral (DV) projections. **(a,c,e,g)** Plain radiographs show indistinct gonads, which are difficult to differentiate from the intestines with indistinct margins from fecal material. **(b,d,f,h)** Contrast-enhanced radiographs reveal well-developed male gonads as radiopaque and elongated patterns in the medial abdominal cavity. Well-developed female gonads appear as radiolucent, rounded oocytes within a radiopaque area in the ventrocaudal abdominal cavity. sb, swim bladder; t, testicle; rt., right testicle; lt, left testicle; ov, ovary; rov, right ovary; lov, left ovary.

In fish with well-developed gonads (Figure 4), the gonads underwent significant enlargement and occupied a major portion of the abdominal cavity. In males, the paired testes extended along the entire length of the cavity, lying ventral to the swim bladder, and each connected posteriorly to a genital duct that opened into the urogenital pore to release spermatozoa into the external environment. Similarly, in females, the paired ovaries extended beneath the swim bladder but were broader than the testes and were covered by the peritoneum only on their medial surfaces. In females, ovaries were distinctly visible, with the contrast medium outlining the spaces between the oocytes. The oocytes appeared as rounded granular structures interspersed within a white background formed by the contrast agent filling the inter-oocyte spaces.

The volume of the contrast agent administered directly influenced the extent of gonadal visualization. Insufficient contrast resulted in partial staining, with limited deposition confined to specific gonadal regions. Conversely, an optimal volume allowed a uniform distribution of the contrast medium, producing comprehensive delineation of the gonads up to their cranial extremities, representing the maximal anatomical boundaries.

## 4 Discussion

In this study, fish were grouped as having less-developed or well-developed gonads based on size, contrast distribution, and necropsy observations. Histological staging was not performed; therefore, these were not definitive reproductive stages. This study demonstrates that contrast-enhanced radiography provides clear *in vivo* visualization of fish gonadal morphology using a simple imaging approach. This is the first report of a non-destructive radiographic technique for detailed fish gonadal imaging. Previous evaluations used ultrasonography, such as in common carp (3) and Nile tilapia, which require a body size of approximately 400 g (17). Conventional radiography in fish is mainly used for species identification (1) and for detecting skeletal abnormalities or foreign bodies (2). The retrograde contrast method expands the diagnostic utility of radiography by visualizing the gonadal position and structure in both male and female fish at different maturation stages.

The success of this approach is closely related to the behavior of the contrast agent within gonadal tissues. Iopamidol, a water-soluble and absorbable contrast medium widely used in cardiology and urinary tract imaging (21, 22), readily entered the reproductive tract after retrograde administration. In males, the contrast medium filled the fine spermatic channels within the testes, generating radiopaque branching patterns that reflected the tubular microarchitecture of the gonads. In females, the contrast medium occupied the interstitial spaces between oocytes, producing a distinct image in which radiopaque areas surrounded relatively radiolucent, rounded oocytes. These differences likely reflect variation in radiographic attenuation among gonadal tissues and contrast-filled spaces (23). Moreover, the oocyte diameter in *Oreochromis niloticus* ranges from 1.78 to 2.48 mm (24), which is sufficient to permit detailed visualization on digital radiographs.

Radiographic patterns provided preliminary qualitative information on gross gonadal development, as reflected by differences in gonadal size, contrast distribution, and necropsy findings. This interpretation is consistent with the endocrine regulation of gonadal development in fish, involving reproductive hormones such as estrogen, progesterone, and testosterone (25), and with progressive gonadal enlargement during reproductive development. In well-developed gonads, the contrast medium was distributed more extensively within the reproductive tract, whereas less-developed gonads showed limited filling, likely due to smaller organ size and less developed ductal spaces. This distinction is relevant in tilapia, where gonadal development may progress rapidly before fish reach market size (26), prompting production strategies such as aquabioponics to better manage growth and reproduction (27, 28). Because ovaries are generally larger than testes at comparable reproductive ages (29), reproductive assessment may be more reliable in females than in males, as reported in ultrasonographic evaluations (30). However, because histological staging, gonadosomatic index, hormonal profiles, and spawning outcomes were not assessed, these findings should not be interpreted as definitive evidence of reproductive maturity.

This study establishes imaging-based reproductive assessment in fish using two-dimensional contrast-enhanced digital radiography. This non-lethal method is a practical alternative to dissection (31) and euthanasia-based gonadal evaluation (32), especially for rare species and aquaculture populations, enhancing selection by identifying individuals with more developed gonadal morphology. This is significant because of the variability in reproductive traits, such as caviar size, among harvested fish (33). In aquatic veterinary medicine, multimodal imaging aids in non-destructive anatomical evaluation (5). Future integration with CT, MRI, or three-dimensional rendering may improve visualization (4, 34). However, findings should be interpreted cautiously. Iopamiro 300 (300 mg iodine/mL) is a non-ionic, water-soluble iodinated contrast medium. Its low-osmolar, non-ionic nature aids tolerability; however, its safety, withdrawal period, tissue residue, and gamete transfer in fish for human consumption are unverified. Thus, iopamidol was used under controlled conditions, and treated fish were not returned to the food chain. Further residue depletion and safety studies are needed before routine use in edible aquaculture species. Although no gross tissue rupture or immediate mortality was observed after contrast agent administration, the physiological effects of retrograde injection are not fully understood, including potential barotrauma, contrast leakage, osmotic stress, and transient tissue irritation. Further validation against histology, gonadosomatic index, oocyte diameter, hormonal profiles, and spawning outcomes is required to confirm diagnostic accuracy, biological safety, and practical suitability before recommending this technique for routine broodstock selection and aquaculture management.

## 5 Limitation

This study was limited by the lack of predetermined reproductive stages and histological validation. Gonadal development was interpreted from radiographic appearance and necropsy confirmation; thus, the maturity categories are descriptive rather than definitive. Future controlled studies should include histology, gonadosomatic index, oocyte diameter, residue analysis, and reproductive outcomes to validate the diagnostic accuracy and biological safety.

## 6 Conclusion

Retrograde positive contrast-enhanced radiography offers a simple method for visualizing gonadal visualization in Nile tilapia. This technique improves the delineation of the testes and ovaries compared with plain radiography and may support non-lethal reproductive assessments in fish. Further validation is needed before routine use in food-producing aquaculture systems.

## Data Availability

The raw data supporting the conclusions of this article will be made available by the authors, without undue reservation.
